# Binocular Stereo Vision-Based Structured Light Scanning System Calibration and Workpiece Surface Measurement Accuracy Analysis

**DOI:** 10.3390/s25206455

**Published:** 2025-10-18

**Authors:** Xinbo Zhang, Li Luo, Rui Ma, Yuexue Wang, Shi Xie, Hao Zhang, Yiqing Zou, Xiaohao Wang, Xinghui Li

**Affiliations:** 1 Tsinghua Shenzhen International Graduate School, Tsinghua University, Shenzhen 518055, Chinamar25@mails.tsinghua.edu.cn (R.M.);; 2 Equipment Technology Research Institute, Liuzhou OVM Machinery Co., Ltd., Liuzhou 545006, Chinazhangh@ovm.cn (H.Z.); zouyq@ovm.cn (Y.Z.)

**Keywords:** structured light, multi-camera calibration, large structural component measurement, measurement accuracy, point cloud stitching

## Abstract

Precise online measurement of large structural components is urgently needed in modern manufacturing and intelligent construction, requiring a measurement range over 1 m, near-millimeter accuracy, second-level measurement speed, and adaptability to complex environments. In this paper, three mainstream measurement technologies, namely the image method, line laser scanning method, and structured light method, are comparatively analyzed. The structured light method exhibits remarkable comprehensive advantages in terms of accuracy and speed; however, it suffers from the issue of occlusion during contour measurement. To tackle this problem, multi-camera stitching is employed, wherein the accuracy of camera calibration plays a crucial role in determining the quality of point cloud stitching. Focusing on the cable tightening scenario of meter-diameter cables in cable-stayed bridges, this study develops a contour measurement system based on the collaboration of multiple structured light cameras. Measurement indicators are optimized through modeling analysis, system construction, and performance verification. During verification, four structured light scanners were adopted, and measurements were repeated 11 times for the test workpieces. Experimental results demonstrate that although the current measurement errors have not yet been stably controlled within the millimeter level, this research provides technical exploration and practical experience for high-precision measurement in the field of intelligent construction, thus laying a solid foundation for subsequent accuracy improvement.

## 1. Introduction

Online precise measurement of the external dimensions and dimensional variations of large structural components is in urgent demand across key fields such as modern manufacturing and intelligent construction [1,2,3]. In industrial manufacturing, the dimensional accuracy of large components directly determines the overall performance and quality of products; for instance, minor dimensional deviations in the large blades of aero-engines may lead to severe issues during high-speed operation. In the field of intelligent construction, such as the construction process of cable-stayed bridges, dimensional monitoring of key bridge structural components serves as a crucial link to ensuring the safety and stability of the bridge. The measurement requirements in these scenarios typically exhibit the following characteristics: a measurement range of over one meter to cover the overall dimensions of large structural components; measurement accuracy approaching the millimeter scale to meet the basic standards of high-precision manufacturing and construction; second-level measurement speed to enable real-time monitoring and rapid feedback in production processes; and adaptability to complex on-site environments to ensure the stability of measurement results. To meet the aforementioned requirements, the current mainstream measurement technologies include the image-based method, line laser scanning method, and structured light method [4,5,6,7,8,9,10,11,12,13,14,15,16,17,18,19,20,21,22,23,24,25,26]. The image-based method primarily relies on computer vision principles, acquiring dimensional information through image capture from multiple angles of the object and subsequent analysis. Its advantages lie in relatively low equipment cost and flexible adaptability to environments. However, this method exhibits limitations in measurement accuracy: especially for large structural components, it struggles to stably achieve the desired high-precision requirements, and its measurement results are susceptible to interference under complex lighting conditions. The line laser scanning method leverages the high directionality and energy density of lasers: it emits a linear laser onto the object surface, captures the reflected light from a specific angle using a camera, and calculates the 3D coordinates of each point on the object surface based on the triangulation principle. This method achieves high measurement accuracy, even reaching the sub-micron level in some precision measurement scenarios. Nevertheless, line laser scanning relies on point-by-point measurement, resulting in relatively slow speed that cannot meet the second-level measurement requirement; additionally, it is sensitive to ambient light, and its measurement accuracy is significantly affected under strong or complex lighting conditions. The structured light method projects specific patterns (e.g., fringes, Gray codes) onto the measured object surface; a camera then captures images of the deformed patterns, and 3D topographic information of the object surface is accurately obtained via algorithms such as phase calculation. This method enables high-precision measurement with fast speed, allowing for the simultaneous acquisition of large-area information on the object surface. However, when measuring the complete contour of complex objects, the structured light method is prone to occlusion issues, leading to missing measurement data in certain regions. Despite these limitations, the structured light method has garnered increasing attention in industries and intelligent construction due to its comprehensive advantages in accuracy and speed.

In practical applications, multi-camera stitching is commonly adopted to address the occlusion issue of the structured light method and achieve full-range measurement of objects. Currently, numerous relevant applications and corresponding key technologies have been proposed. For example, some studies have improved the measurement accuracy and stability of multi-camera systems by optimizing camera layout and calibration algorithms [27,28,29,30,31,32,33,34,35]; other works have focused on enhancing the design and decoding algorithms of structured light patterns to improve adaptability to complex scenarios [36,37,38,39,40]. These methods provide valuable references for multi-camera stitching measurement. However, due to differences in hardware configurations, measurement principles, and application scenarios among various structured light systems, camera calibration becomes extremely complex, and calibration results vary significantly across systems. As a core link in multi-camera structured light measurement systems, camera calibration accuracy plays a decisive role in the subsequent point cloud stitching quality. High-precision calibration ensures the accurate alignment of point cloud data captured by different cameras in the spatial coordinate system, thereby enabling complete and precise 3D reconstruction of the object. If the calibration accuracy meets a certain standard, point cloud stitching can even be achieved solely through coordinate transformation, without relying on feature point matching. This advantage holds great significance in practical applications: it significantly reduces the time cost required for point-cloud stitching and minimizes stitching errors caused by inaccurate feature point matching.

In view of this, the present study focuses on the practical scenario of cable tensioning for meter-diameter cables in cable-stayed bridges within the context of intelligent construction, and conducts research on a contour measurement method and system based on the collaboration of multiple structured light cameras. In this scenario, real-time measurement of the cable diameter changes during tensioning is crucial for ensuring the installation quality and structural safety of the bridge. According to practical test results, although the current measurement error has not yet been fully and stably controlled within the 1 mm range, the study optimizes measurement indicators through a series of efforts: conducting modeling analysis of the measurement system to refine parameter configurations; constructing the measurement system by selecting appropriate hardware (e.g., structured light cameras, projectors) and completing system integration; and performing performance verification to continuously evaluate and optimize key performance indicators (KPIs) such as measurement accuracy, speed, and stability, with the goal of constantly moving closer to the millimeter-level accuracy requirement. Through these efforts, this study aims to provide new technical exploration and practical experience for high-precision measurement in intelligent construction and related industrial fields, laying a foundation for further improving measurement accuracy in subsequent studies.

## 2. Materials and Methods

### 2.1. Measurement System Hardware and Configuration

Four identical structured light 3D scanners were employed as the core sensing units, each integrating a stereo vision module (one industrial camera) and a structured light projector (capable of emitting fringe and Gray-code patterns).

**Figure 1:**
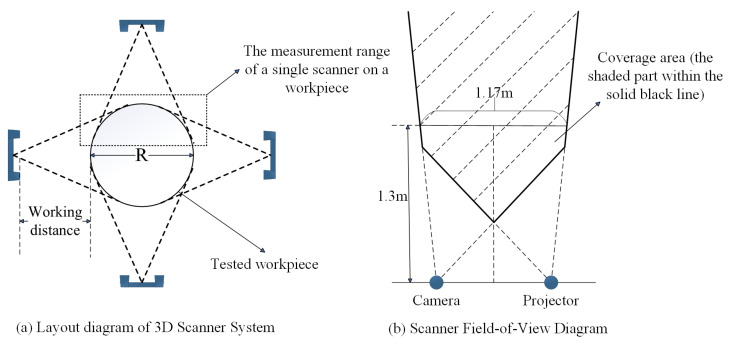
Layout and FOV of four structured light scanners.

The scanners were symmetrically distributed around the circular test workpiece (Figure 1a), with the workpiece idealized as a cylinder with radius R, while the instantaneous measurement volume of each scanner is represented by a triangular footprint. Four scanners are arranged symmetrically distributed around the cylinder axis; by carefully setting the working distance—defined as the perpendicular separation between the foremost optical interface of the scanner and the cylindrical surface—a multi-view scanning network is established. The field-of-view of a single scanner, indicated by the black dashed rectangle in the figure, covers a localized region of the cylindrical surface; through mutual compensation of the individual perspectives, the array enables full-circumference 3D reconstruction of the workpiece.

Figure 1b illustrates the field of view (FoV) of a single structured-light 3D scanner in detail. The core sensing module comprises a camera and a projector: the projector emits a patterned fringe sequence onto the target surface, while the camera synchronously acquires the correspondingly modulated images. The overlapping region of the two optical cones—highlighted as the shaded area within the solid black contour—defines the valid measurement volume. Within this domain, the complete fringe set is recorded by the camera, enabling the internal algorithms (phase unwrapping and stereo matching) to reconstruct the three-dimensional geometry of the object.

**Figure 2:**
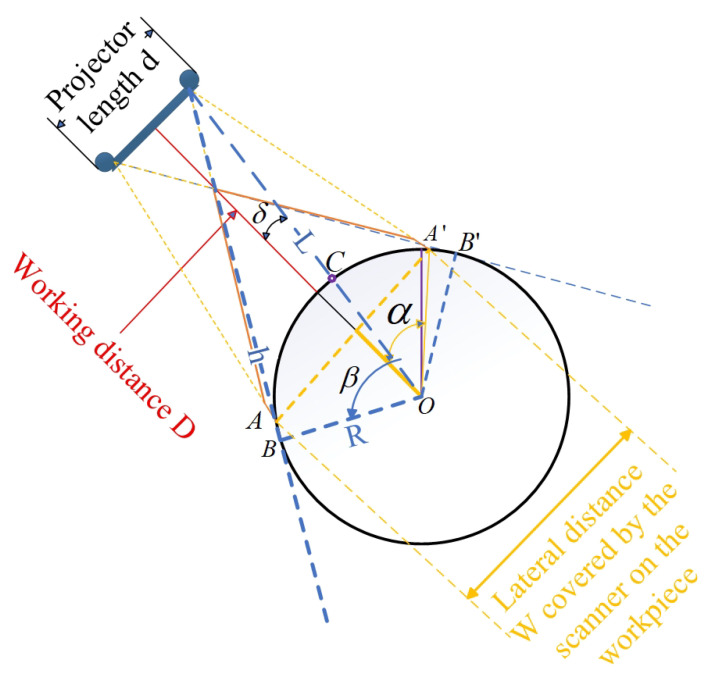
Coverage angle and curvature analysis of a single scanner.

Figure 2 illustrates the comprehensive coverage capability of a single 3D structured light scanner for curved workpieces from the perspective of geometrical optics, including the coupling relationship between the curvature of the workpiece surface and the scanning field of view (FOV). As shown in the figure, the orange solid line represents the maximum coverage range of the scanner, while the tangent position *B* or $B^{\prime }$ of the blue dashed line tangent to the curved surface determines whether the workpiece will be occluded at the front and rear ends relative to the scanner within the effective range of the scanner, causing point cloud loss at the occluded area. The main parameters in the figure are as follows: α represents the angle value corresponding to half of the arc length $\overset{⌢}{AA^{\prime }}$ of the workpiece area that the scanner’s FOV can cover; β represents the angle value corresponding to the arc $\overset{⌢}{A^{\prime }C}$ or $\overset{⌢}{AC}$ between the tangent point *B* of the camera’s FOV and the edge of the workpiece and the intersection point *C* of the line connecting the camera and the workpiece center *O* with the curved surface; δ is an angular parameter introduced for the convenience of geometric analysis and has no actual physical significance. As shown in Figure 2, if β−δ is greater than α, no occlusion will occur; otherwise, occlusion will occur. *d* represents the length of the 3D structured light scanner, *D* represents the working distance (the perpendicular distance from the foremost end of the scanner to the workpiece surface), *R* represents the radius of the circular workpiece, *L* represents the linear distance between the camera and the workpiece center (which can be obtained by the Pythagorean theorem), and *h* represents the length of the line segment with the tangent point between the camera and the edge of the workpiece as the endpoint. *W* represents the lateral distance covered by the scanner’s FOV on the workpiece. From the Pythagorean theorem and relevant knowledge of plane geometry, the calculation formulas for α, β, and *L* can be easily derived as follows.

(1) $$\alpha =\arcsin\left(\frac{W}{2R}\right)$$

(2) $$\beta =\arccos\left(\frac{R}{L}\right)$$

(3) $$L=\sqrt{{\left(D+R\right)}^{2}+{\left(\frac{d}{2}\right)}^{2}}$$

(4) $$\delta =\arccos\left(\frac{D+R}{L}\right)$$

Two types of workpiece were used to validate the accuracy of the measurement of the system, with a priorly known surface areas (serving as the ground-truth reference):

- Cylindrical-like workpiece: a hollow plastic cylinder simulating the regular contour of stay cables in cable-stayed bridges, with a true CSA (cross sectional area) of $1.81581\;m^{2}$.
- Irregular annular workpiece: a metal annular component simulating the irregular contour of aged cables, with a true CSA of $1.75569\;m^{2}$.

**Figure 3:**
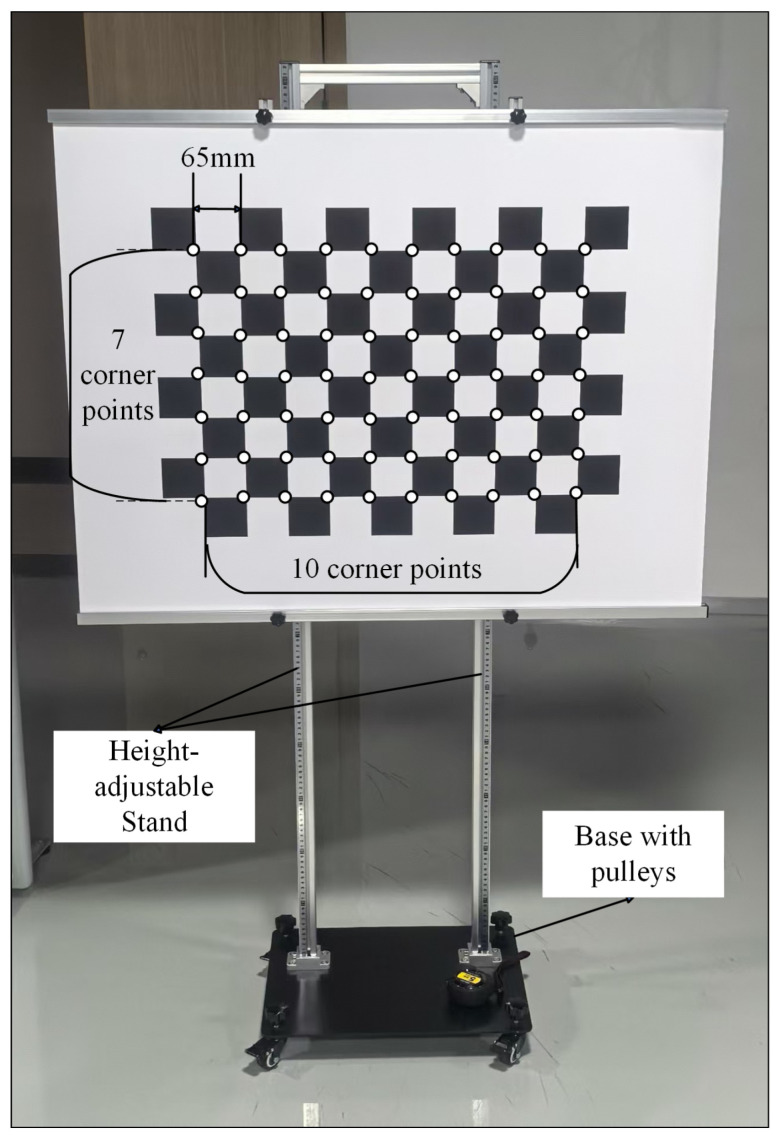
Physical image of the chessboard calibration board.

A high-precision chessboard calibration board (Figure 3) was utilized to establish the correspondence between 3D world points and 2D image points, a prerequisite for camera intrinsic and extrinsic calibration.

- Specifications: the board featured a 10×7 corner array (10 horizontal corners, 7 vertical corners) with an inter-corner physical distance of 65mm.
- Corner Detection: subpixel-level corner coordinates were extracted using computer vision algorithms (e.g., OpenCV functions), matching the known 3D world coordinates of the corners (with the calibration board plane defined as Z=0 in the world coordinate system).

### 2.2. Multi-Scanner Calibration and Coordinate Transformation

**Figure 4:**
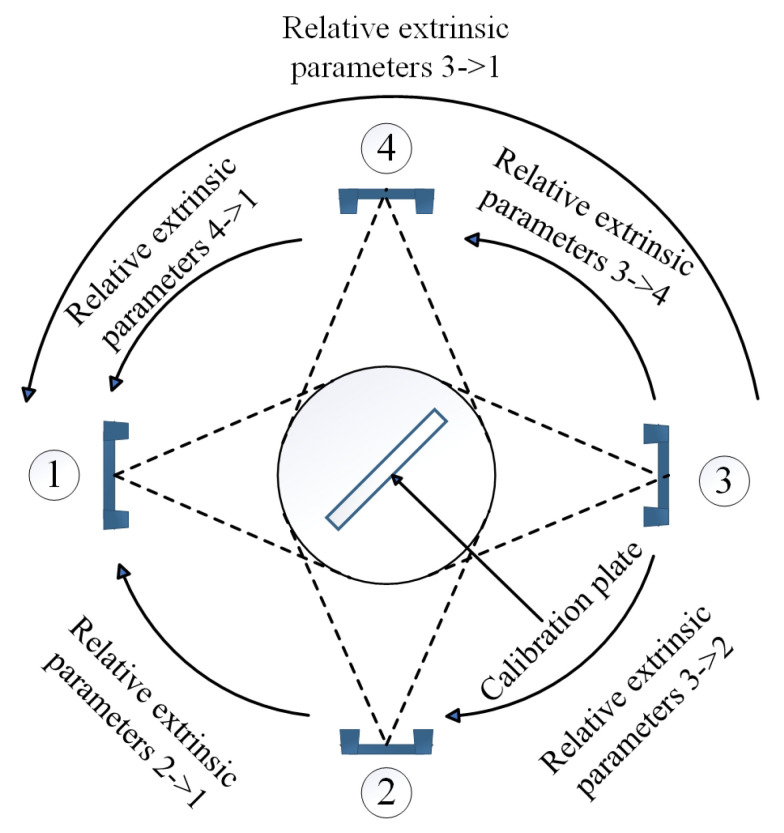
Schematic of the calibration process.

To unify the coordinate systems of the four scanners (numbered ①–④), a global calibration strategy based on relative extrinsic parameters was proposed (Figure 4), addressing the limited overlapping FOV between Scanner ① and ④ (which prevented direct acquisition of high-quality calibration images):

1. Calibrate Scanner ① and ③ to compute the relative extrinsic matrix $T_{2\to 1}$ (containing rotation matrix $R_{2\to 1}$ and translation vector $t_{2\to 1}$), establishing their pose relationship.
2. Calibrate Scanner ① and ④ to obtain $T_{4\to 1}$, linking Scanner ④ to the reference frame of Scanner ①.
3. Calibrate Scanner ③ and ④ to derive $T_{3\to 4}$, defining Scanner ③’s pose relative to Scanner ④.
4. Calculate $T_{3\to 1}$ (Scanner ③ to ①) via matrix chain multiplication: $T_{3\to 1}=T_{3\to 4}\cdot T_{4\to 1}$, where $R_{3\to 1}=R_{3\to 4}\cdot R_{4\to 1}$, and $t_{3\to 1}=t_{3\to 4}+R_{3\to 4}\cdot t_{4\to 1}$.

**Figure 5:**
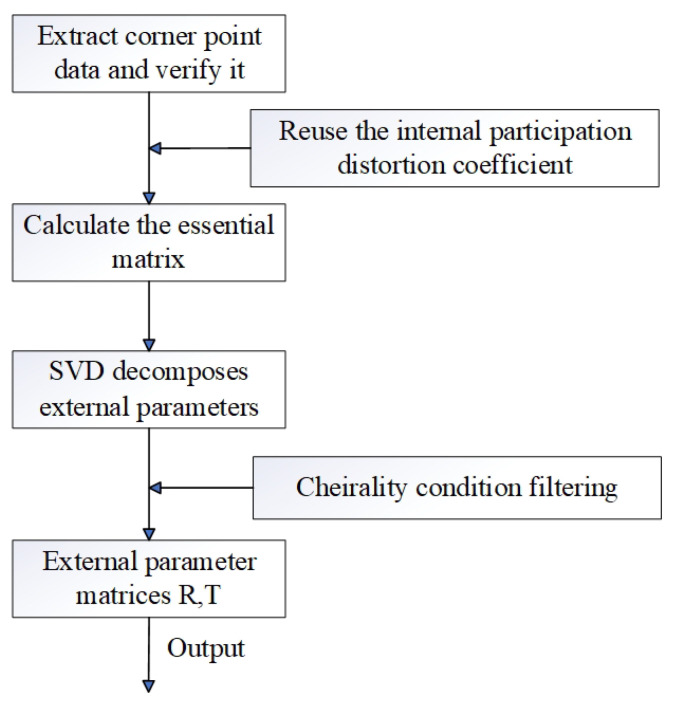
Logic of the calibration algorithm.

After calibration, all scanners’ coordinates were unified under Scanner ①’s camera coordinate system (defined as the global reference frame). The calibration algorithm logic (Figure 5) included corner data verification, intrinsic parameter reuse, essential matrix calculation, and singular value decomposition (SVD) for extrinsic parameter extraction.

**Figure 6:**
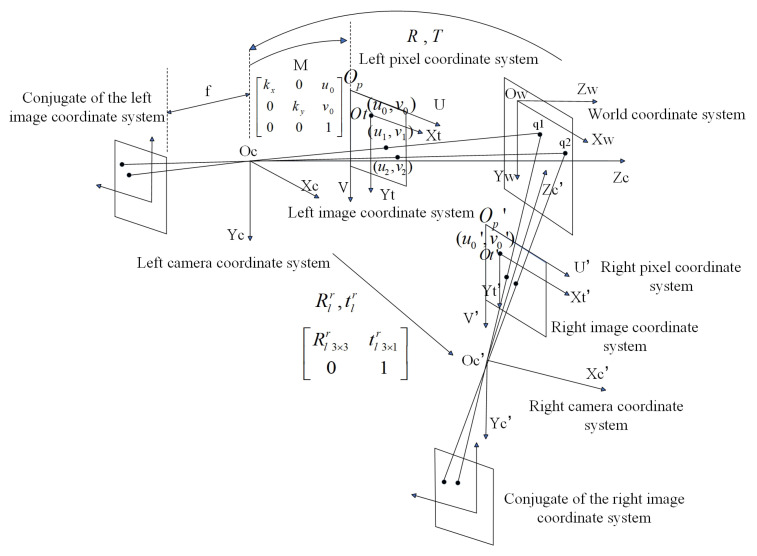
Schematic of coordinate transformation in stereo calibration.

The mapping from 3D world points to 2D pixel points involved seven coordinate systems (Figure 6), namely the world coordinate system $O_{w}-X_{w}Y_{w}Z_{w}$ (a global reference system that describes the absolute position of the 3D scene), the left camera coordinate system $O_{c}-X_{c}Y_{c}Z_{c}$ (a local reference system of the left camera with its origin at the optical center of the left camera), the left image coordinate system $O_{t}-X_{t}Y_{t}$ (a pixel-level coordinate system of the left image, with its origin being the projection point of the origin of the world coordinate system onto the left image coordinate system), the left pixel coordinate system $O_{p}-UV$ (a pixel-level coordinate system of the left image, with its origin at the upper-left corner of the image), the right camera coordinate system $O_{c}^{\prime }-X_{c}^{\prime }Y_{c}^{\prime }Z_{c}^{\prime }$ (a local reference system of the right camera, with its origin at the optical center of the right camera), the right image coordinate system $O_{t}^{\prime }-X_{t}^{\prime }Y_{t}^{\prime }$ (a millimeter-level coordinate system of the right image, with its origin being the projection point of the origin of the world coordinate system onto the right image coordinate system), and the right pixel coordinate system $O_{p}^{\prime }-U^{\prime }V^{\prime }$ (a pixel-level coordinate system of the right image with its origin at the upper-left corner of the image).

#### 2.2.1. Key Coordinate Transformations

**Figure 7:**
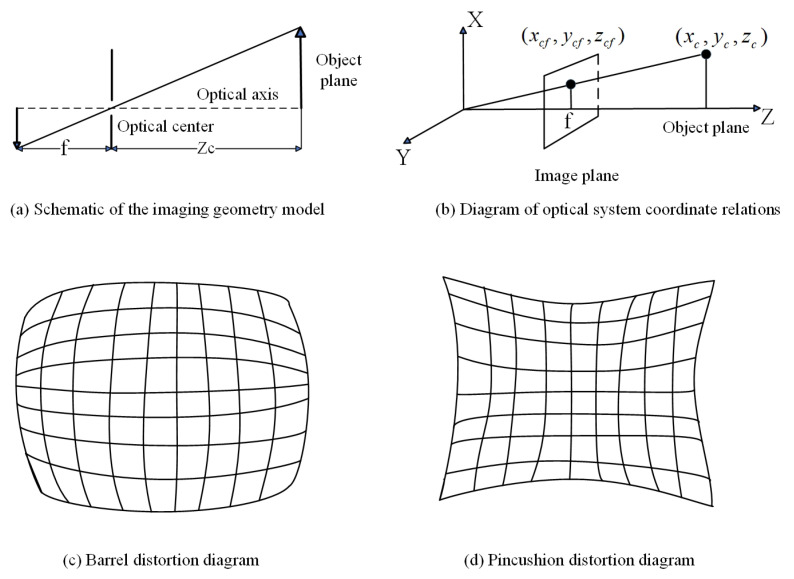
Schematic of perspective projection and radial distortion.

1. World → Camera Coordinates: Taking the left scanner as the analysis object, there exists a transformation matrix from the world coordinate system to the left camera coordinate system [41]. Then, we have
  (5) $$\left[\begin{array}{c} x_{c}\\ y_{c}\\ z_{c}\\ 1 \end{array}\right]=\left[\begin{array}{cc} R & t\\ 0 & 1 \end{array}\right]\left[\begin{array}{c} x_{w}\\ y_{w}\\ z_{w}\\ 1 \end{array}\right],$$
  where R (3 × 3) and t (3 × 1) are the rotation and translation parameters of the scanner relative to the world frame.
2. Camera → Image Coordinates: This process realizes the conversion from three-dimensional coordinates to two-dimensional coordinates [42]. The imaging process is shown in Figure 7a. For the convenience of description, we swap the positions of the camera coordinate system and the image coordinate system, flip the image, and equivalent a erect virtual image at a distance equal to the front focal length from the optical center, resulting in the arrangement shown in Figure 7b.
  From Figure 7b, we can obtain
  (6) $$\left\{\begin{array}{c} \frac{x_{cf}}{f}=\frac{x_{c}}{z_{c}}\\ \frac{y_{cf}}{f}=\frac{y_{c}}{z_{c}} \end{array}\right.,$$
  where *f* is the camera’s focal length.
3. Image → Pixel Coordinates: The coordinate of the origin of the image coordinate system in the pixel coordinate system is $\left(u_{0},v_{0}\right)$. The physical dimensions of each pixel in the *x*-axis and *y*-axis directions of the image coordinate system are $\alpha_{x}$ and $\alpha_{y}$, and the coordinate of the image point in the actual image coordinate system is $\left(x_{\mathrm{cf}},y_{\mathrm{cf}}\right)$.
  Converting to the homogeneous coordinate representation form, we can get the mapping relationship from the world coordinate system to the pixel coordinate system:
  (7) $$\begin{array}{cc} \left[\begin{array}{c} u\\ v\\ 1 \end{array}\right] & =\frac{1}{z_{c}}\left[\begin{array}{ccc} f\alpha_{x} & 0 & u_{0}\\ 0 & f\alpha_{y} & v_{0}\\ 0 & 0 & 1 \end{array}\right]\left[\begin{array}{cc} R & t\\ 0 & 1 \end{array}\right]\left[\begin{array}{c} x_{w}\\ y_{w}\\ z_{w}\\ 1 \end{array}\right]\;\left(\mathrm{let}\;z_{w}=0\right)\\ \; & =\frac{1}{z_{c}}\left[\begin{array}{ccc} f\alpha_{x} & 0 & u_{0}\\ 0 & f\alpha_{y} & v_{0}\\ 0 & 0 & 1 \end{array}\right]\left[\begin{array}{cccc} r_{1} & r_{2} & r_{3} & t\\ 0 & 0 & 0 & 1 \end{array}\right]\left[\begin{array}{c} x_{w}\\ y_{w}\\ z_{w}\\ 1 \end{array}\right]\\ \; & =\frac{1}{z_{c}}\left[\begin{array}{ccc} f_{x} & 0 & u_{0}\\ 0 & f_{y} & v_{0}\\ 0 & 0 & 1 \end{array}\right]\left[\begin{array}{ccc} r_{1} & r_{2} & t\\ 0 & 0 & 1\\ 0 & 0 & 1 \end{array}\right]\left[\begin{array}{c} x_{w}\\ y_{w}\\ 1 \end{array}\right],\;\left(\mathrm{where}\;\left[\begin{array}{ccc} r_{1} & r_{2} & t\\ 0 & 0 & 1\\ 0 & 0 & 1 \end{array}\right]\left[\begin{array}{c} x_{w}\\ y_{w}\\ 1 \end{array}\right]=\left[\begin{array}{c} x_{c}\\ y_{c}\\ 1 \end{array}\right]\right) \end{array}.$$
  In Equation (7), distortion is not included in the formula, but it does not prevent the use of distortion coefficients for correction in the actual process.

#### 2.2.2. Distortion Correction

From the ideal image coordinate system to the actual image coordinate system (distortion needs to be considered) [43], radial and tangential distortions are most significant; so, we only consider radial and tangential distortions here.

Radial distortion is caused by the manufacturing process of the lens. The two types of radial distortion are shown in Figure 7c and Figure 7d: barrel distortion and pincushion distortion, respectively.

Tangential distortion is caused by the installation position errors of the lens and CMOS or CCD.

Therefore, the overall distortion correction formula is as follows.

(8) $$\left[\begin{array}{c} x_{cf}^{\prime }\\ y_{cf}^{\prime } \end{array}\right]=\left(1+k_{1}r^{2}+k_{2}r^{4}\right)\left[\begin{array}{c} x_{cf}\\ y_{cf} \end{array}\right]+\left[\begin{array}{c} 2p_{1}x_{cf}y_{cf}+p_{2}\left(r^{2}+2x_{cf}^{2}\right)\\ 2p_{2}x_{cf}y_{cf}+p_{1}\left(r^{2}+2y_{cf}^{2}\right) \end{array}\right].$$

In the formula, the first term represents the radial distortion, and the second term represents the tangential distortion, where $k_{1}$, $k_{2}$ are mirror distortion correction coefficients, and $p_{1}$, $p_{2}$ are tangential distortion correction coefficients $r=\sqrt{x^{2}+y^{2}}$.

#### 2.2.3. The Solution Principle of Stereo Calibration

Our ultimate goal is to find the relative external parameters $R^{\prime }$ and $t^{\prime }$ between the left and right cameras. The solution principle of binocular calibration is as follows:

Let us assume that there is a point *a* whose coordinate in the camera coordinate system is *A* and whose coordinates in the left and right pixel coordinate systems are $a_{1}$ and $a_{2}$ respectively. The internal parameter matrices are $K_{1}$ and $K_{2}$. Therefore, we can formulate

(9) $$\left\{\begin{array}{c} a_{1}=K_{1}A\\ a_{2}=K_{2}\left(R^{\prime }A+t^{\prime }\right) \end{array}\right..$$

Let $x_{1}=k_{1}^{-1}a_{1}$, $x_{2}=k_{2}^{-1}a_{2}$; then, $x_{2}=R^{\prime }x_{1}+t^{\prime }$. After some derivation, we obtain

(10) $$x_{2}^{T}t^{\prime \wedge }R^{\prime }x_{1}=0.$$

Let $E=t^{\prime \wedge }R^{\prime }$, and *E* is the essential matrix, which is the basis for solving the relative external parameters later.

#### 2.2.4. Verification of Full Coverage

**Figure 8:**
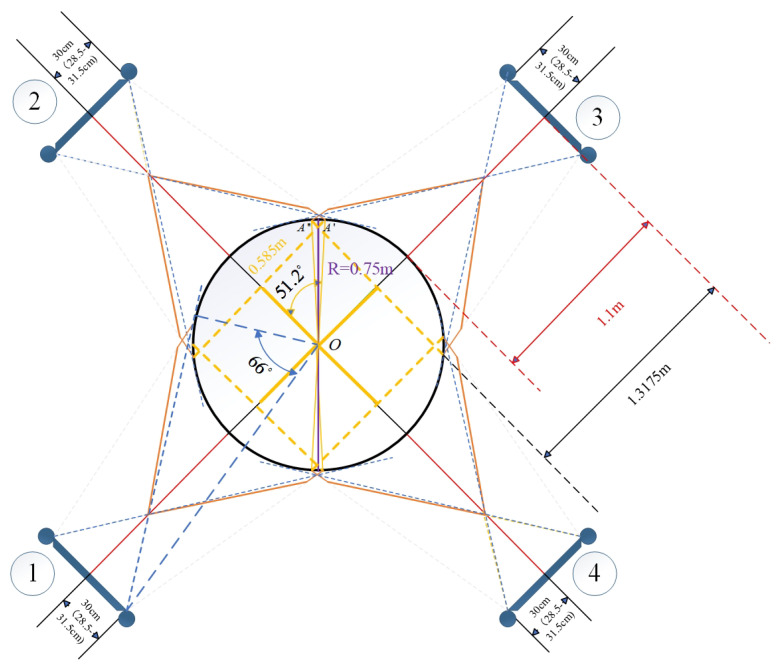
Actual coverage of the four scanners.

The actual coverage of the four scanners on the cylindrical workpiece is shown in Figure 8 (a specific case of Figure 2 under experimental parameters). The sizes of various key parameters in this experimental environment are shown in Table 1 below.

Substituting the key parameters in Table 1 into Equations (1)–(4), we can get L≈1.875m, α=51.2°, β≈66°, and δ=9.36°. Since β−δ>α, it can be judged that the curvature factor of the workpiece surface will not affect the coverage range of the camera.

**Figure 9:**
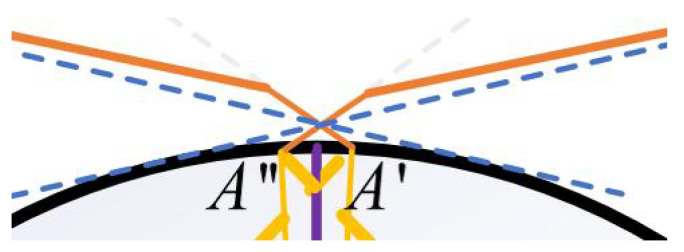
Local magnification of FOV overlap.

Local magnification of the FOV overlap between Scanner ② and ③ (Figure 9) confirmed that the edge points of each scanner’s FOV fell within the adjacent scanner’s FOV, ensuring redundant coverage and no data loss.

### 2.3. 3D Contour Measurement and Data Processing

#### 2.3.1. Measurement Process

1. Structured Light Projection and Image Acquisition: Each scanner’s projector emitted coded patterns (fringes) onto the workpiece surface. The structured light scanning system utilizes a camera from Luster Inc, ShenZhen, China. (model: Y2000L), equipped with an 8 mm focal length lens and a CMOS photoreceiver matrix with 1624 × 1240 resolution and 4.5 µm/pixel pixel size. The stereo cameras synchronously captured deformed pattern images within the effective FOV (Figure 1b), with data acquired via custom software (developed using Visual Studio 2022 and OpenCV 4.11).
2. Local 3D Reconstruction: The scanner’s internal algorithm performed phase unwrapping and stereo matching on the captured images to generate local 3D point clouds of the workpiece surface.
3. Global Point Cloud Stitching: Local point clouds from the four scanners were transformed into the global reference frame (Scanner ①’s coordinate system) using the calibrated relative extrinsic parameters $T_{i\to 1}$ (i=2,3,4). High calibration accuracy enabled stitching via direct coordinate transformation, eliminating the need for feature point matching.

**Figure 10:**
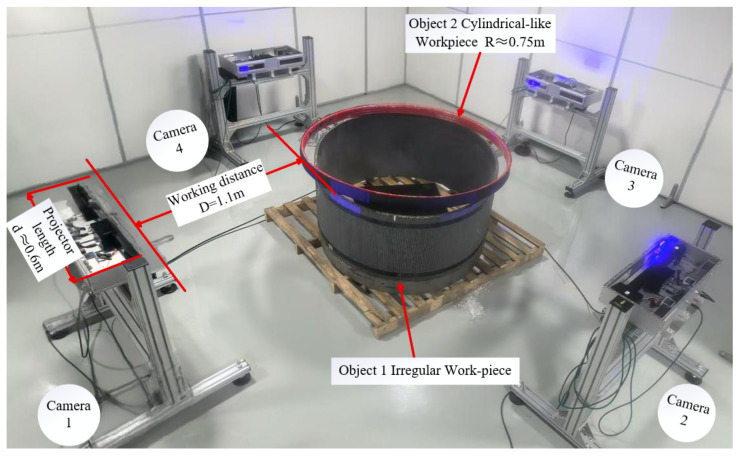
Physical image of the four scanners and test workpieces.

Figure 10 shows the physical image of the four scanners and test workpieces.

#### 2.3.2. Data Validation and Error Analysis

1. Experimental Protocol: The measurement was repeated 11 times for both workpieces to assess stability. For each trial, the workpiece diameter was computed by fitting the stitched point cloud to an irregular annular contour.
2. Error Calculation: the relative error was defined as
  Relative Error = $\frac{\left|\mathrm{Measured}\;\mathrm{CSA}-\mathrm{True}\;\mathrm{CSA}\right|}{\mathrm{True}\;\mathrm{CSA}}\times 100\%$.
  The measurement results (including measured values, true values, and relative errors) are summarized in Table 2.
3. Statistical Analysis: Quartile analysis (upper limit, 75th percentile, median, 25th percentile, lower limit) and outlier detection (interquartile range, IQR method) were conducted. No outliers were identified (IQR = 0.032 for the irregular workpiece, IQR = 0.044 for the cylindrical-like workpiece), confirming the system’s reliability.

### 2.4. Ethical Statement and Data Availability

- Ethical Approval: ethical review and approval were waived for this study, as it involved only inanimate industrial workpieces and no human or animal subjects.
- Data Availability: the experimental data supporting the findings—including scanner calibration parameters, raw image datasets, reconstructed point clouds, and measurement results—are available from the corresponding author upon reasonable request.
- Software and Tools: the measurement system was developed using Visual Studio 2022 (Microsoft, Redmond, WA, USA) and OpenCV 4.11 (Open Source Computer Vision Library, https://opencv.org/releases/, (accessed on 3 July 2025)).

## 3. Results

### 3.1. Verification of Full Coverage by Four Scanners

To confirm that the four structured light scanners could achieve complete circumferential coverage of the cylindrical-like workpiece (radius R=0.75m) without blind spots, geometric analysis and experimental validation were conducted.

Substituting the key system parameters (Table 1) into Equations (1)–(4) yielded the following critical angles:

- Coverage angle α=51.2° (half the arc angle covered by a single scanner);
- Constraint angle β=66.4° (angle determining potential occlusion);
- Angle δ=9.36° (angular parameter introduced for the convenience of geometric analysis and has no actual physical significance).

Since β−δ>α, the workpiece curvature did not block the scanner’s FOV, confirming no local data loss from a single scanner.

**Figure 11:**
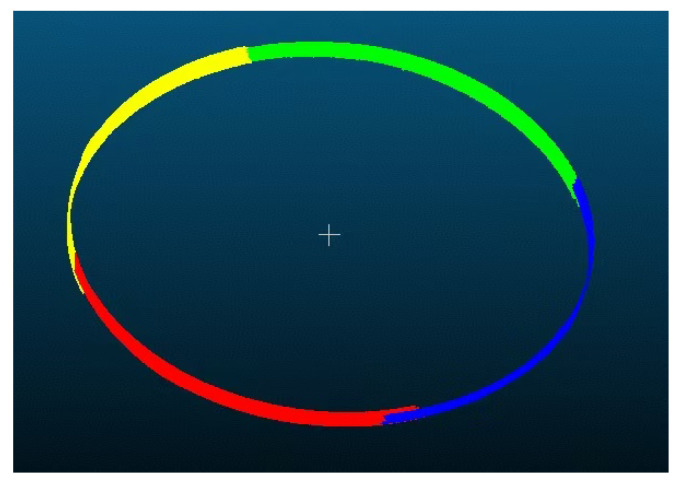
Point cloud overlap images acquired by the actual scanner.

Figure 11 illustrates the actual coverage of the four scanners on the cylindrical workpiece in a real experimental environment. It visually shows that the FOVs of adjacent scanners (e.g., Scanner ① and ②, ③ and ④) overlap by approximately 12.4°, ensuring no gaps in circumferential coverage.

For further verification, Figure 9 provides a local magnification of the FOV overlap between Scanner ② and ③. The edge point of Scanner ②’s FOV on the workpiece surface fell within Scanner ③’s FOV and vice versa. This redundant coverage confirmed that the four scanners collectively achieved full circumferential reconstruction of the workpiece without blind spots.

### 3.2. Measurement Accuracy of the System

The system’s measurement accuracy was evaluated using two types of workpieces (cylindrical-like and irregular annular) with known true CSA. Each workpiece was measured 11 times, and the results are summarized in Table 2.

1. Measurement Results for Cylindrical-like Workpiece

- True CSA: 1.81581 m^2^;
- Measured CSA range: 1.809097–1.810775 m^2^;
- Relative error range: 0.277–0.370% (corresponding to an absolute error of D about the range of 2.1–2.8 mm);
- Median relative error: 0.345% (absolute error: 2.6 mm).

2. Measurement Results for Irregular Annular Workpiece

- True CSA: 1.75569 m^2^;
- Measured CSA range: 1.751210–1.752639 m^2^;
- Relative error range: 0.174–0.255% (corresponding to an absolute error range of 1.1–2.1 mm);
- Median relative error: 0.241% (absolute error: 1.8 mm).

Notably, the irregular workpiece exhibited lower relative errors, which was attributed to its rougher surface enhancing structured light pattern modulation—unlike the smooth surface of the cylindrical-like workpiece, which caused slight specular reflection and reduced pattern contrast.

### 3.3. Statistical Analysis of Measurement Stability

To assess the system’s stability, quartile analysis and outlier detection (using the Interquartile Range, IQR method) were performed on the 11 sets of measurement data.

The quartile statistics for relative errors are shown in Table 3.

Outlier analysis was conducted using the IQR criterion (outliers defined as values outside [Q1−1.5×IQR,Q3+1.5×IQR]):

- For the irregular workpiece: IQR = 0.032%, range [0.166%,0.294%]; all 11 relative errors fell within this range, with 0 outliers.
- For the cylindrical-like workpiece: IQR = 0.044%, range [0.246%,0.422%]; all 11 relative errors were within this range, with 0 outliers.

These results confirmed the system’s high stability, with no abnormal fluctuations in repeated measurements.

## 4. Discussion

### 4.1. Interpretation of Measurement Results

The experimental results demonstrate that the multi-structured light system achieves stable circumferential coverage and measurable accuracy for large-scale cylindrical-like components. The key findings and their implications are discussed below.

The geometric calculation (β−α>δ) and experimental validation (Figure 1 and Figure 10) confirm that the four scanners’ symmetric layout (Figure 1a) effectively avoids occlusion caused by workpiece curvature. This addresses the core limitation of single-structured light systems (local data loss) and provides a feasible solution for the full-contour measurement of large cylindrical components (e.g., stay cables of cable-stayed bridges).

The system’s relative errors (0.174–0.370%) are higher than the target of “millimeter-level absolute error” (1 mm), primarily due to two factors:

- Distortion Residuals: Although radial and tangential distortions were corrected using Equations (8), residual distortion (<0.3 pixels) from lens manufacturing defects (Figure 5) led to an absolute error of ∼2.1 mm in 3D reconstruction. This residual distortion error is systematic, as it stems from the inherent approximation of the distortion model and introduces consistent bias in measurements.
- Calibration Error: For each camera-pair calibration (using the shared calibration board), we captured 15 images with varying calibration board poses. The calibration error is bounded by reprojection error: the reprojection error for all camera pairs is less than 0.7 pixels. The chained calibration for Scanner ① and ③ (Figure 4) introduced a cumulative error in relative pose estimation, as matrix chain multiplication amplifies small errors from intermediate steps (Scanner ④’s calibration error).

Notably, the irregular workpiece’s lower error (0.174–0.255%) highlights the system’s better adaptability to rough surfaces; this is because structured light patterns are more effectively modulated by rough surfaces, improving the stereo matching accuracy.

The absence of outliers (Section 3.3) and narrow percentile range confirm the system’s high stability, which benefits from two aspects:

1. Hardware Synchronization: since the measured object was static in the experiment, the scanners were set to acquire asynchronously to avoid mutual interference between structured light patterns.
2. Algorithm Robustness: the calibration algorithm (Figure 5) included corner data verification and SVD-based extrinsic decomposition, which reduced the impact of noise (e.g., uneven lighting) on parameter estimation.

### 4.2. Current Limitations

1. Environmental Sensitivity: the system’s accuracy decreases and may even lead to point-cloud dropouts under strong ambient light, as excessive light washes out structured light patterns, which limits outdoor applications (e.g., on-site cable-stayed bridge measurements).
2. Calibration Complexity: the chained calibration for non-overlapping scanners (① and ③) requires 15–20 sets of calibration images, which is time-consuming (approximately 40 min per system).

### 4.3. Future Perspectives

1. Anti-Glare Design: integrate a narrow-band filter (450 nm, matching the scanner’s projection wavelength) to reduce ambient light interference, enabling outdoor use.
2. Fast Calibration: develop a dynamic calibration method using a portable reference sphere to reduce calibration time to <10 min.
3. Error Compensation: introduce a laser interferometer to measure residual errors and establish a compensation model, aiming to further reduce absolute error (approaching the millimeter-level target).
4. Refined calibration: use higher-precision calibration targets (e.g., ceramic-coated chessboards with sub-micron flatness) and increase the calibration image count to reduce residual systematic errors.

## 5. Conclusions

This study developed a multi-structured light contour measurement system for large cylindrical components (e.g., stay cables of cable-stayed bridges) and validated its performance through experiments. The key conclusions are as follows:

1. Coverage Capability: The four scanners’ symmetric layout (1.1 m working distance) and optimized FOV design achieve full circumferential coverage of cylindrical workpieces (radius 0.75 m) without blind spots, as confirmed by geometric calculations (β−α>δ) and experimental validation (overlapping FOVs of adjacent scanners).
2. Measurement Performance: The system exhibits stable and measurable accuracy: for irregular annular workpieces: relative error 0.174–0.255% (absolute error 1.1–2.1 mm); for cylindrical-like workpieces: relative error 0.277–0.370% (absolute error 2.1–2.8 mm); no outliers in 11 repeated measurements, confirming high stability.
3. Practical Value: The system addresses the occlusion limitation of single-structured light systems and reduces hardware costs by using only four scanners. It provides a feasible technical solution for the real-time contour measurement of large cylindrical components in intelligent construction (e.g., cable tensioning monitoring of cable-stayed bridges), laying a foundation for future millimeter-level accuracy optimization.

## Figures and Tables

**Table 1:** Key parameter values of the system.

Key Parameter	Parameter Value
Working distance *D*	1.1 m
Projector length *d*	Approximately 60 cm (57–63 cm)
Workpiece radius *R*	0.75 m
Lateral distance *W* covered by the scanner on the workpiece	1.17 m

**Table 2:** Measurement results.

Serial Number	Cylindrical-Like (m^2^)	True Value (m^2^)	Relative Error (%)	Irregular Workpiece (m^2^)	True Value (m^2^)	Relative Error (%)
1	1.809687	1.81581	0.337	1.752301	1.75569	0.193
2	1.809538	1.81581	0.345	1.751374	1.75569	0.246
3	1.809525	1.81581	0.346	1.751741	1.75569	0.225
4	1.809287	1.81581	0.359	1.751441	1.75569	0.242
5	1.809097	1.81581	0.370	1.752639	1.75569	0.174
6	1.809341	1.81581	0.356	1.751210	1.75569	0.255
7	1.809393	1.81581	0.353	1.751405	1.75569	0.244
8	1.810142	1.81581	0.312	1.751234	1.75569	0.253
9	1.810102	1.81581	0.314	1.751868	1.75569	0.218
10	1.810147	1.81581	0.312	1.751464	1.75569	0.241
11	1.810775	1.81581	0.277	1.751931	1.75569	0.214

**Table 3:** Statistical distribution of measurement relative errors.

Statistic	Irregular Annular Workpiece%	Cylindrical-Like Workpiece%
Upper Limit	0.255	0.370
75th Percentile	0.246	0.356
Median	0.241	0.345
25th Percentile	0.214	0.312
Lower Limit	0.174	0.277

The narrow range of percentiles (e.g., 0.081% for the irregular workpiece, 0.093% for the cylindrical-like workpiece) indicated consistent measurement results across repeated trials.

## Data Availability

Datasets generated during the current study are available from the corresponding authors on reasonable request.
